# Modulation of Macrophage Polarization by Phospholipids on the Surface of Titanium

**DOI:** 10.3390/molecules25112700

**Published:** 2020-06-10

**Authors:** Hongxuan Quan, Yongjoon Kim, Lele Wu, Hee-Chul Park, Hyeong-Cheol Yang

**Affiliations:** Department of Dental Biomaterials Science, Dental Research Institute and BK21 Plus Program, School of Dentistry, Seoul National University, 101 Daehak-ro, Jongno-gu, Seoul 03080, Korea; baobei@snu.ac.kr (H.Q.); yjak87@snu.ac.kr (Y.K.); 2018-32117@snu.ac.kr (L.W.); pipichul@snu.ac.kr (H.-C.P.)

**Keywords:** phospholipid, phosphatidylserine, macrophage, polarization, titanium

## Abstract

Macrophage polarization has become increasingly important for the improvement of the biocompatibility of biomaterials. In this study, we coated Ti discs with phospholipids (phosphatidylserine/phosphatidylcholine [4:1 mole/mole]) by evaporating the solvent under vacuum, and observed the polarization of RAW 264.7 cells cultured on the discs. The coated discs were hydrated before cell culture was added. The shape of the hydrated phospholipids varied with the concentration of loaded phospholipids: a perforated layer (0.1 mM), tubules and spheres (1 mM), and spheres (10 mM). RAW 264.7 cells exhibited different morphologies, depending on the concentration of phospholipids. On the coated discs, the gene expression and protein release of TGF-β1, VEGF, Arg-1, and TNF-α were downregulated, especially with 10 mM phospholipids. The stimulation of mRNA expression and the protein release of those genes by IL-4 and LPS were also disturbed on the phospholipid-coated discs. In conclusion, the polarization of RAW 264.7 cells was prevented by hydrated phospholipids on Ti discs.

## 1. Introduction

Tissue injury is inevitably caused by surgical procedures for the implantation of medical devices. The occurrence of tissue injury is accompanied by inflammation and immune responses, which include the formation of a provisional matrix, coagulation, the appearance of neutrophils and macrophages, granulation tissue formation, and tissue remodeling [1]. The degree and duration of immune responses are affected by the size, biodegradability, toxicity, and surface topography of the implanted biomaterials [2]. From the viewpoint of cellular reactions, the chemical and physical properties of biomaterials may influence the activity of various types of immune cells. Among the cells related to the immune response, macrophages are considered key cells that characterize the tissue response to biomaterials because of their phenotypic diversity, along with multiple functions [3]. The phenotype of polarized macrophages can be divided into M1 (classically activated) and M2 (alternatively activated) subsets [4]. M1 macrophages secrete a high level of pro-inflammatory cytokines such as tumor necrosis factor-α (TNF-α) and interleukin-1β (IL-1β), and intensify the inflammatory tissue reaction. In contrast M1 macrophages, M2 macrophages are known to release anti-inflammatory cytokines and promote the resolution of inflammation and wound healing.

The activation and phenotype of macrophages is likely affected by implanted biomaterials, and the resulting state of macrophages may, in turn, determine the quality of tissue regeneration on the surface of the biomaterials. Since the beginning of biomaterial design, there have been many attempts to regulate the state of macrophages to improve their biocompatibility [5,6,7]. To enhance the biocompatibility of biomaterials, M2 polarization of macrophages is generally recommended, because anti-inflammatory M2 macrophages are supposed to end inflammation and promote tissue regeneration. Meanwhile, the suppression of macrophage activation has been mostly studied in the pharmaceutical field to treat inflammatory diseases. M2-inducing cytokines have been widely used to generate M2 macrophages around biomaterials. IL-4, a representative M2-inducing cytokine, loaded in gelatin methacryloyl hydrogel and polypropylene mesh successfully promoted the polarization of macrophages to M2 in in vitro and in vivo experiments [8,9]. IL-10 also changed macrophage phenotype that promoted muscle regeneration [10]. Thus, it is certain that those cytokines are effective for M2 polarization followed by tissue regeneration. However, the application of the M2-inducing cytokines is often limited by other clinical risks due to their multiple biological functions. For example, IL-4 is known to promote several types of tumor [11], and to play a role in allergies and autoimmune diseases [12,13]. Therefore, the clinical use of cytokines for M2 induction should be carefully deliberated.

The modulation of macrophage behavior was also added to dental and orthopedic Ti implants to improve osseointegration. The hydrophilized surface of Ti implants exhibited accelerated early osseointegration in a previous clinical study [14]. The increase in hydrophilicity was found to suppress the release of pro-inflammatory cytokines and promote M2 macrophage polarization [15,16]. The nanostructure of the Ti surface also induced M2 activation in vitro and in vivo, and exhibited higher bone-to-implant contact compared to the group with the smooth surface [17]. Those results imply that M2 polarization is advantageous for the osseointegration of Ti implants.

To modulate macrophage activation, phosphatidylserine (PS) -mimicking apoptotic cells have been used in a form of liposomes or as a coating material [7,18]. PS, exposed on the surface of apoptotic cells, can bind to macrophage PS receptors and induce macrophages to release anti-inflammatory cytokines such as transforming growth factor (TGF)-β [19]. Therefore, Ti surfaces coated with PS are also expected to induce M2 macrophage polarization and influence osseointegration. In fact, a supported lipid bilayer containing PS induced M2-like polarization of the murine macrophage cell line, RAW 264.7, on Ti surfaces [20]. Besides the formation of the lipid bilayer, Ti has been coated with PS-containing phospholipids through the evaporation of an organic solvent and the deposition of phospholipids on the Ti surface [18,21]. The evaporation method is much easier than the formation of a supported lipid bilayer for the coating of the phospholipid, and the coating obtained by evaporation can be preserved in dry conditions, while aqueous conditions are necessary to maintain the supported lipid bilayer. The evaporation method is also thought to be applicable to various biomedical devices unless the surface of devices was dissolved by organic solvents. However, the effect of the evaporation-deposited PS-phospholipid on macrophage polarization has not yet been fully investigated. In this study, we examined the morphology of a hydrated phospholipid that was deposited on a Ti surface by evaporation of organic solvent, and the effect of the phospholipid on macrophage polarization was assessed to inquire whether the coating methods of PS-phospholipid affect the cellular properties of macrophages.

## 2. Materials and Methods

### 2.1. Phospholipid Coating of Ti Discs

Machined discs of commercially pure titanium (grade 2, diameter 20 mm, thickness 0.5 mm) were obtained from SEOUL TITANIUM Co., Ltd. (Seoul, Korea). PS was purchased from Avanti Polar Lipids (Alabaster, Al, USA). All other experimental reagents were purchased from Sigma-Aldrich Co. (Saint Louis, MO, USA) unless otherwise specified. The machined Ti discs were cleaned with chloroform, acetone, ethanol, and distilled water, and then autoclaved. PS and phosphatidylcholine (PC) were dissolved in chloroform/methanol (9:1 *v*/*v*) at 2.5 and 7.5 mM, respectively. The phospholipid mixture solution (10 mM total concentration) was then diluted with chloroform/methanol to prepare the 1.0 and 0.1 mM solutions. To coat the Ti discs, 50 µL of undiluted and diluted phospholipid solutions were loaded onto the surface of the discs, and the organic solvent was evaporated in a vacuum chamber for 2 h. Finally, the surface was rehydrated by immersion in culture medium for 6 or 24 h before cell culturing. After hydration, the phospholipid suspension was removed by gentle shaking, to leave only phospholipids adhered on the discs.

### 2.2. RAW 264.7 Cell Culture and Cell Polarization

RAW 264.7, a murine macrophage cell line, was obtained from RIKEN Cell Bank (Tsukuba, Japan), and maintained in Dulbecco’s modified Eagle’s medium (DMEM) (WELGENE Inc., Gyeongsan, Korea) containing 10% heat-inactivated fetal bovine serum (Gibco-BRL, Grand Island, NY, USA) and 1% antibiotics (100 U/mL penicillin-G and 100 mg/mL streptomycin) at 37 °C in a humidified atmosphere (5% CO_2_/95% Air). To culture the RAW 264.7 cells on Ti discs, 1 mL of cell suspension containing 5 × 10^5^ cells was added on a Ti disc placed in a 12-well plate. To induce M1 and M2 RAW 264.7 cell phenotypes, 50 ng/mL lipopolysaccharide (LPS) (E. coli, serotype 055:B5) and 40 ng/mL IL-4 were added to the culture, respectively, after stabilizing the cells on Ti discs for 1 h.

### 2.3. Scanning Electron Microscopy

The morphology of the phospholipid coats and macrophages on the Ti discs was observed with a field emission scanning electron microscope (FE-SEM, Hitachi S-4700, Hitachi Ltd., Tokyo, Japan). The samples were fixed with 2.5% glutaraldehyde in 0.1 M phosphate buffer and dehydrated with ethanol. A conformal gold-palladium coating was applied with a Polaron-range sputter coater. The surface morphology was observed under the FE-SEM at an acceleration voltage of 15 kV. The number of phospholipid spheres was counted manually from three SEM images (25.5 × 19 µm^2^), and the size of the spheres was measured using the imageJ software (National Institute of Health, Bethesda, MD, USA).

### 2.4. Analysis of Gene Expression in Macrophages

Gene expression of TGF-β1, vascular endothelial growth factor (VEGF), Arginase-1 (Arg-1), and TNF-α were analyzed by quantitative polymerase chain reaction (qPCR). Six hours after depositing the cell culture on the Ti surface, total RNA was isolated using an RNA isolation reagent (WellPrep Total RNA Isolation Reagent, Welgene Inc., Daegu, Korea), and cDNA was prepared from total RNA using a power cDNA Synthesis Kit (iNtRON Biotechnology, Sungnam, Korea). PCR was performed in a 20 μL mixture containing 10 μL SYBR Premix Ex Taq (Takara Bio, Otsu, Japan), 0.4 μL ROX Reference Dye (Takara Bio), cDNA, and primers, using an ABI PRISM 7500 (Applied Biosystem, Carlsbad, CA, USA). The sequences of the primers used are as follows: TGF-β1, forward: 5′-TGGAGCAACATGTGGAACTC-3′, reverse: 5′-TGCCGTACAACTCCAGTGAC-3′; VEGF, forward: 5′-TTACTGCTGTACCTCCACC-3′ reverse: 5′-ACAGGACGGCTTGAAGATG-3′; Arg-1, forward: 5′-CAAGAAGAATGGAAGAGTCAG-3′, reverse: 5′-CAGATATGCAGGGAGTCACC-3′; TNF-α, forward: 5′-GGCAGGTCTACTTTGGAGTCATTGC-3′, reverse: 5′-ACATTCGAGGCTCCAGTGAATTCGG-3′; Gyceraldehyde 3-phosphate dehydrogenase (GAPDH), forward: 5′-TGTGTCCGTCGTGGATCTGA-3′, reverse: 5′-CCTGCTTCACCACCTTCTTGAT-3′. Thermo-cycling conditions were 95 °C for 30 s, followed by 40 cycles of denaturation at 95 °C for 15 s and annealing at 60 °C for 1 min. All targeted cytokine expression levels were calculated based on their threshold cycle (CT) values, and were expressed as relative mRNA expression ratios normalized to a reference gene (GAPDH).

### 2.5. Enzyme-Linked Immunosorbent Assay (ELISA)

The release of TGF-β1, VEGF, Arg-1, and TNF-α protein by RAW 264.7 cells on the phospholipid-coated Ti surface was analyzed by ELISA. After culture for 24 h, cell culture supernatants were collected, and the amount of protein was determined with an ELISA kit (Quantikine HS, R&D systems Inc., Minneapolis, MN, USA). The absorbance at 450 nm was measured with a microplate reader (Sunrise, TECAN, Salzburg, Austria).

### 2.6. Fluorescence Microscopy for Observation of Phospholipid and Actin Structure

The phospholipids used for Ti coating were visualized with a fluorescence probe, N-(6-tetramethyl-rhodamine-thiocarbamoyl)-1,2-dihexadecanoyl-sn-glycero-3-phosphatidyl ethanol-amine (TRITC-DHPE, Biotium Inc., Hayward, CA, USA). Then, 0.5 mol% TRITC-DHPE was added to the phospholipid mixture before loading onto Ti discs.

To observe the actin structure, the cells were fixed with 4% paraformaldehyde and permeabilized with 0.5% Triton X-100 for 15 min. The samples were further incubated in 1% BSA for 1 h to block nonspecific interactions and stained with ActinGreen^™^ 488 ReadyProbes^®^ (Thermo Fisher Scientific, Waltham, MA, USA). Mounting medium with 4′, 6-diamidino-2-phenylindole (DAPI) (ab104139, Abcam, Cambridge, MA, USA) was then added to visualize the nucleus.

### 2.7. Statistical Analysis

All data were obtained from three independent experiments and expressed as the mean ± standard deviation (SD). Differences among the groups were assessed by one-way analysis of variance (one-way ANOVA) followed by Tukey’s test. Statistical analyses were performed using SPSS 22 statistical software (SPSS, Chicago, IL, USA). *p*-values less than 0.05 were considered statistically significant.

## 3. Results

### 3.1. Surface Morphology of Phospholipid-Coated Ti

**Figure 1:**
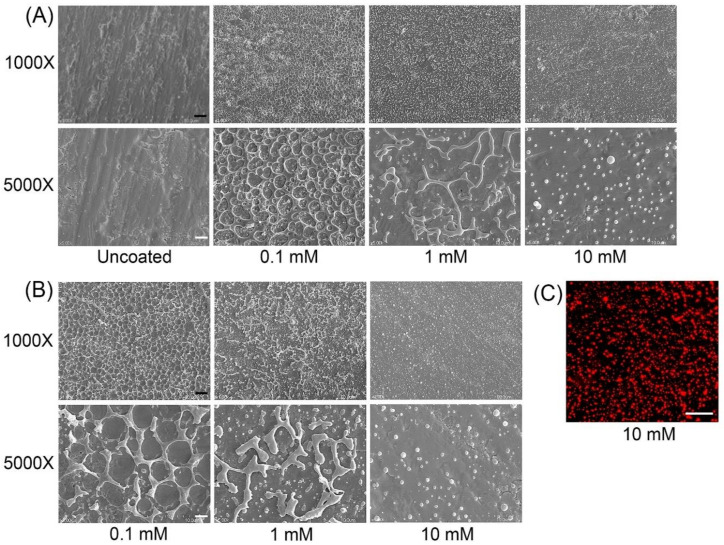
SEM (**A**,**B**) and fluorescence (**C**) images of phospholipid coats on Ti discs. Ti discs were coated with phospholipids by loading 0.1, 1, and 10 mM phospholipid solution, followed by drying and hydration for 6 (**A**) and 24 h (**B**). Black (**A**) and white (**B**) bars indicate 10 and 2 μm, respectively. White bar in (**C**) indicates 10 μm.

Phospholipid film has been commonly used for the preparation of liposomes [22,23]. During the process of liposome production, dried lipid film absorbs water by hydration and then swells and forms various shapes on the solid surface. Therefore, the phospholipid film on Ti discs may swell under water unless Ti inhibits the hydration of lipids. In this study, we coated Ti discs with various amounts of phospholipids and observed the hydrated surface using SEM. As shown in Figure 1A, discs loaded with 0.1 mM of phospholipids exhibited a randomly perforated thin layer after hydration for 6 h. When the disc was coated with 1 mM of phospholipids, amorphous tubules and spherical structures appeared. With 10 mM of phospholipids, only spherical structures were formed. The spheres with diameters ranging between approximately 100 and 1500 nm were dispersed evenly over the surface, and 3.6 ± 0.46 spheres, on average, adhered to the 10 μm^2^ surface area.

After hydration for 24 h, the tubular structures mostly grew in size or thickness (1.0 mM phospholipid), and the holes in the perforated layer became larger on the 0.1-mM-phospholipid-coated discs compared to 6 h-hydrated phospholipids (Figure 1B). However, on the Ti surface loaded with 10 mM of phospholipids, there was no significant difference in size and spherical morphology between the groups of 6 h and 24 h hydration. Furthermore, the number of spheres remaining on the surface was not affected by hydration time. The spherical structure of phospholipids was also confirmed by the labeling of phospholipids with fluorescent TRITC-DHPE (Figure 1C). Those results indicated that 10 mM of phospholipids could give rise to a stable spherical structure of phospholipids on machined Ti surfaces, even after longer periods of hydration.

### 3.2. Morphology of RAW 264.1 Cells on the Phospholipid-Coated Ti Surface

**Figure 2:**
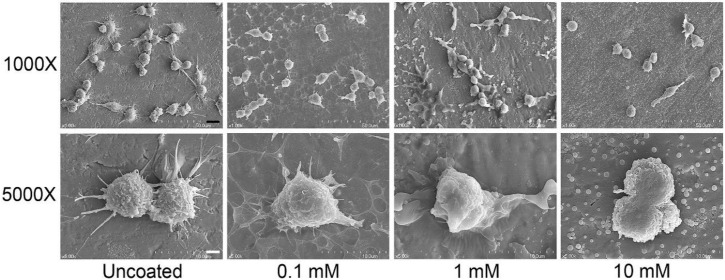
SEM images of RAW 264.7 cells cultured on phospholipid-coated Ti discs. Cells were cultured on Ti discs for 24 h. Before cell culture, phospholipid-coated Ti discs were prepared using the same method (6 h of hydration) as described in Figure 1. Black and white bars indicate 10 and 2 μm, respectively.

The morphology of RAW 264.7 cells on the phospholipid-coated Ti surface was observed by SEM (Figure 2).

Cells were cultured for 24 h on 6 h hydrated discs. On an uncoated disc, various kinds of cell morphology were found: round, oval-shaped, and elongated. Most cells had a considerable number of filopodia. On the disc with a 0.1 mM phospholipid coating, oval- and round-shaped cells with filopodia were observed on the perforated layer of phospholipids. When the cells were cultured on 1 mM phospholipid-coated discs, empty spaces devoid of phospholipids were frequently seen, and the cell surface looked smoother than those on the uncoated discs. It was likely that the cells became entangled with phospholipid structures during cell culture. On the discs prepared with 10 mM phospholipids, the cells were rounder and less elongated. Some cells did not exhibit the protrusion of filopodia. Many phospholipid spheres still remained on the surface of the discs. Therefore, the most obvious difference in cell morphology was seen between the cells on uncoated and 10-mM phospholipid-coated discs.

### 3.3. Polarization-Regulated Gene Expression and Protein Release of RAW 264.7 Cells on Phospholipid-Coated Ti Discs

**Figure 3:**
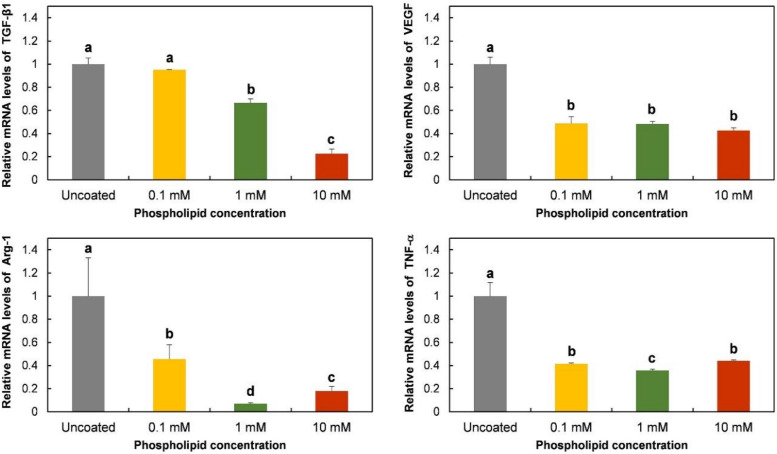
Gene expression of RAW 264.7 cells cultured on phospholipid-coated Ti discs. Ti discs were previously coated with 0.1, 1, or 10 mM of phospholipids and hydrated for 6 h. On the discs, cells were cultured for 6 h to determine the relative expression of genes encoding TGF-β1, VEGF, Arg-1, and TNF-α by qPCR analysis. The data are presented as the mean ± SD, and different letters (**a**, **b**, **c**, and **d**) above the error bars indicate statistically significant differences (*p* < 0.05). Values sharing a letter are not significantly different.

Gene expression of TGF-β1, VEGF, Arg-1, and TNF-α was evaluated to observe the effects of phospholipid coating on macrophage polarization. TNF-α, a pro-inflammatory protein, is known to be expressed in M1 phenotypes, while TGF-β1, VEGF, and Arg-1 are strongly or specifically expressed in M2 type cells [24]. As shown in Figure 3, the expression of the TGF-β1 gene was inhibited by a 10 mM phospholipid coating, compared to the expression level on uncoated Ti discs. The inhibitory effect disappeared on 0.1 mM lipid-coated discs. The phospholipid coating also suppressed the mRNA expression of VEGF, Arg-1, and the TNF-α gene. The expression of those genes was prevented at all tested concentrations.

**Figure 4:**
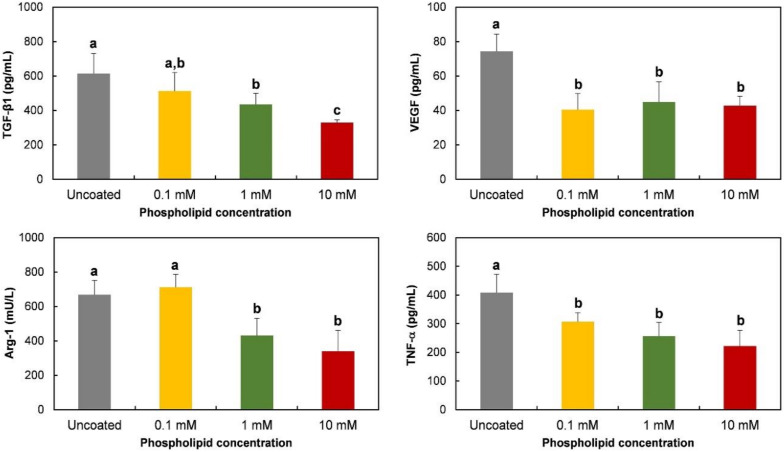
Amount of released protein from RAW 264.7 cells cultured on phospholipid-coated Ti discs. Cells were cultured for 24 h on the same Ti discs of Figure 3. TGF-β1, VEGF, Arg-1, and the TNF-α protein in supernatants were quantified with ELISA. The data are presented as the mean ± SD, and different letters (**a**, **b**, and **c**) above the error bars indicate statistically significant differences (*p* < 0.05). Values sharing a letter are not significantly different.

The protein release of TGF-β1, VEGF, Arg-1, and TNF-α was also observed on phospholipid-coated Ti discs. The tendency of protein release was mostly consistent with the gene expression shown in Figure 4. The release of TGF-β1 was inhibited dose-dependently, while VEGF was suppressed to the same level at all tested phospholipid concentrations. As expected with the mRNA assessment, Arg-1 and TNF-α protein were also suppressed on the coated discs. Therefore, it was obvious that smaller amounts of pro- and anti-inflammatory proteins were released by RAW 264.7 cells on Ti discs that were coated with phospholipids.

### 3.4. Gene Expression and Protein Release of RAW 264.7 Cells on Phospholipid-Coated Ti Discs under Polarization-Inducing Conditions

**Figure 5:**
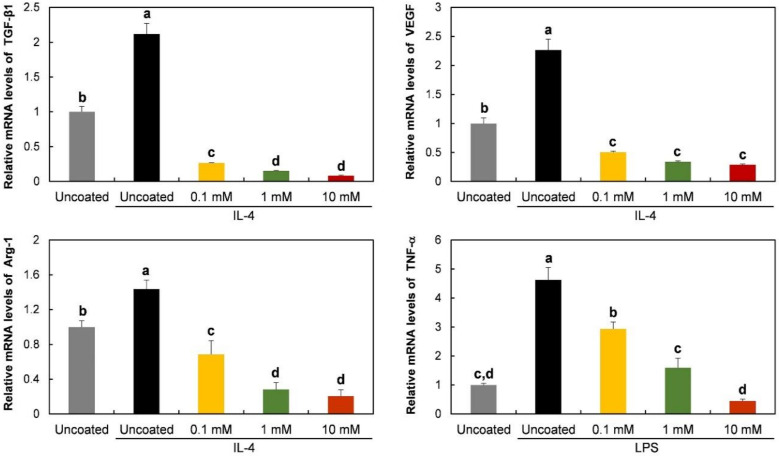
Gene expression of stimulated RAW 264.7 cells cultured on phospholipid-coated Ti discs. To stimulate the cells, RAW 264.7 were exposed to IL-4 (40 ng/mL) or LPS (50 ng/mL) for 6 h on Ti discs, and gene expression was analyzed as seen in Figure 3. The data are presented as the mean ± SD, and different letters (**a**, **b**, **c**, and **d**) above the error bars indicate statistically significant differences (*p* < 0.05). Values sharing a letter are not significantly different.

mRNA expression and protein release were observed in the presence of polarization-inducing agents. As shown in Figure 5, on the uncoated discs, TGF-β1, VEGF, and Arg-1 were upregulated by IL-4, an M2-inducing agent, during culture for 6 h. However, the enhancement of the mRNA expression of those anti-inflammatory genes by IL-4 was significantly inhibited by the phospholipid coating. Moreover, the expression levels on the coated discs were lower than those of unstimulated cells on uncoated discs. To induce the M1 phenotype, the cells were exposed to LPS. TNF-α mRNA level was increased by LPS on uncoated discs, and the LPS effect was blocked by phospholipids on the Ti discs. The blocking effect of phospholipids appeared dose-dependently. Only 10 mM of phospholipids completely prevented the effect of LPS.

**Figure 6:**
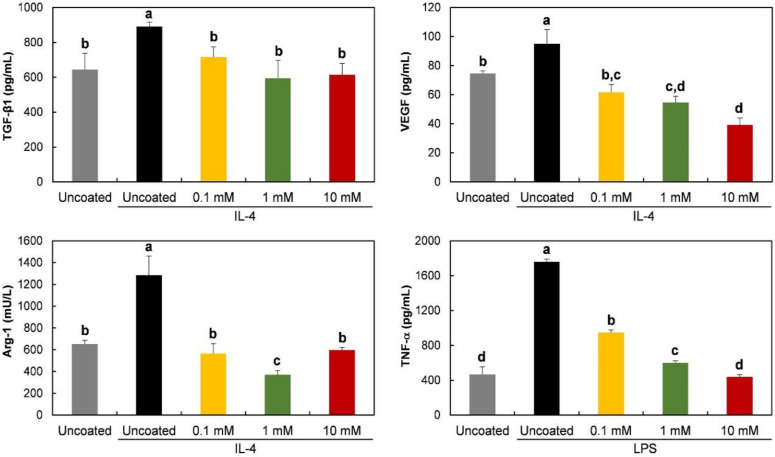
Amount of released protein from stimulated RAW 264.7 cells cultured on phospholipid-coated Ti discs. To stimulate the cells, RAW 264.7 were exposed to IL-4 (40 ng/mL) or LPS (50 ng/mL) for 24 h on Ti discs, and TGF-β1, VEGF, Arg-1, and the TNF-α protein in supernatants were quantified with ELISA. The data are presented as the mean ± SD, and different letters (**a**, **b**, **c**, and **d**) above the error bars indicate statistically significant differences (*p* < 0.05). Values sharing a letter are not significantly different.

In accord with mRNA expression, the release of TGF-β1, VEGF, and the Arg-1 protein was enhanced by IL-4. The enhancing effect of IL-4 was blocked on phospholipid-coated-Ti discs (Figure 6). The LPS-induced increase in TNF-α release was also inhibited by the phospholipid coating, as shown in Figure 6. Ten mM of phospholipids caused the strongest blocking effect on protein release.

### 3.5. Actin Structure of RAW 264.7 Cells on Phospholipid-Coated Ti Discs

**Figure 7:**
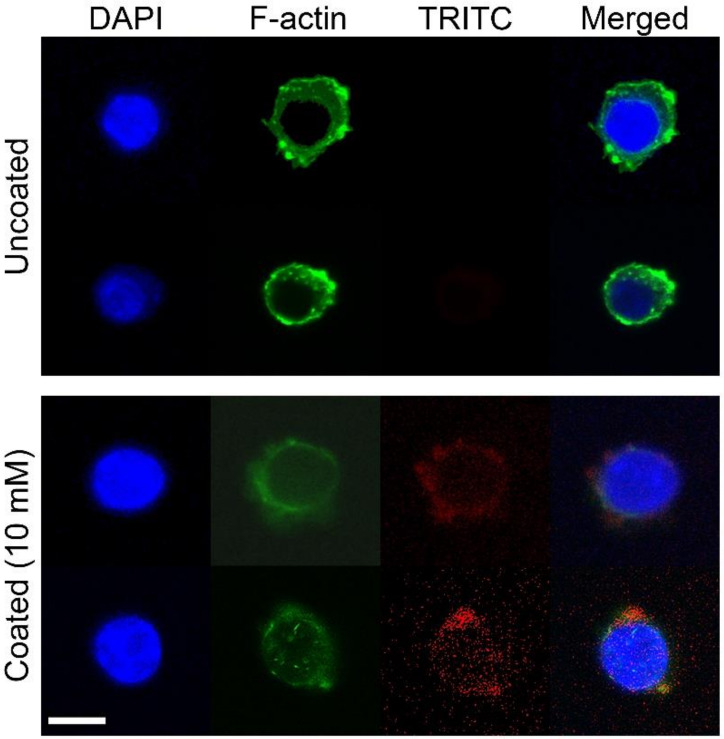
Fluorescence images of RAW 264.7 cells cultured on phospholipid-coated Ti discs. Cells were cultured on phospholipid (10 mM)-coated Ti discs for 6 h, and were stained with DAPI and ActinGreen^™^ 488 ReadyProbes^®^ to visualize the nucleus (blue) and F-actin (green), respectively. Phospholipids were previously labeled with TRITC-DHPE (red) before Ti coating. White bar indicates 5 μm.

The activation and polarization of macrophages is normally accompanied with changes in cell shape where the modification of actin structures clearly occurs [25]. Therefore, an altered actin structure was expected by culture on phospholipid-coated discs, because the phospholipid coating induced changes in cell morphology (Figure 2) and inflammatory responses (Figure 3 and Figure 4). On the uncoated discs, filamentous actin (F-actin) was clearly visible at the edges of the cell membranes, indicating that actin assembly was actively occurring beneath the plasma membrane (Figure 7). However, many macrophages exhibited faint fluorescence with less intensity at the cellular edges on phospholipid (10 mM)-coated discs. It was likely that the actin dynamics interfered with the phospholipids on the Ti discs. In addition to the actin structure, TRITC-DHPE was also observed to track phospholipids during cell culture.

As shown in Figure 7, TRITC-DHPE fluorescence spread out within the cell body, and no specific intracellular localization was observed when using the probe. The fluorescence inside the cells indicated that the phospholipids on the Ti discs were phagocytosed or absorbed by RAW 264.7 cells.

## 4. Discussion

A PS-containing supported phospholipid bilayer was bound to induce the M2 phenotype of RAW 264.7 cells on a Ti surface [20]. It was theorized that PS polarizes the macrophage-like cells by binding to the PS receptors, mimicking the interaction between apoptotic cells and macrophages. Therefore, in this study, we expected that the RAW 264.7 cells would express M2 properties on the phospholipid-coated Ti surface because of the presence of PS in phospholipids. However, on the contrary, the gene expression and protein release of Arg-1, an M2-specific cytokine, was downregulated on the phospholipid-coated Ti discs (Figure 3). Furthermore, the gene expression of TGF-β and VEGF, favored by the M2 phenotype, was also inhibited by phospholipid coating compared to the uncoated discs. Those results suggest that PS did not function as an ‘eat-me’ signal which can induce the M2 phenotype of macrophages. The inhibitory action of phospholipids was not limited to the expression of the M2 phenotype. TNF-α, an M1 specific cytokine, was also inhibited by the phospholipid coating on a transcription level (Figure 3). Therefore, it is likely that the macrophages were not heading for either the M1 and M2 phenotypes, but rather, becoming more inactive on the phospholipid-coated Ti surface. Cell morphology supported our speculation about the state of the macrophages. Cells on the phospholipid-coated surface, especially 10 mM phospholipids, presented fewer cell membrane extensions, such as filopodia (Figure 2). Since inactive RAW 264.7 cells are generally round with less protrusion of the membrane [25], cells on the phospholipid-coated surface in Figure 2 seemed to be more inactive than those on uncoated discs. The tendency of cells to be inactive on the phospholipid-coated surface appeared more clearly in the experiments with polarization-inducing agents. M2-inducing IL-4 could not stimulate the expression of TGF-β, VEGF, and Arg-1, and the M1-inducing activity of LPS was inhibited by the phospholipid coating (Figure 5 and Figure 6). These results imply that phospholipids on the Ti surface actively interfered with macrophage activation. The differences of macrophage activation on the supported lipid bilayer and the phospholipid coating of this study suggest that the coating method, as well as the coating materials, are critical factors in determining cellular properties.

Various phospholipid shapes appeared on the Ti surface after hydration (Figure 1). The morphology varied among different concentrations of phospholipids. At higher concentrations, smaller-sized lipids were produced. Completely isolated and spherical liposome-like structures were formed with 10 mM of phospholipids, even after 24 h of hydration. It was likely that the formation of liposome-like structures was slower at lower phospholipid concentrations, which happened to lead to the formation of connected tubular structures. The mechanism underlining the relationship between morphology and concentration remains unknown. As described previously, macrophage activation was interfered with on phospholipid-coated cells, even though PS, an M2-inducing phospholipid, was included in the phospholipids. Since PS-contained phospholipid, as a form of a supported bilayer, induced M2 polarization on Ti discs, the inhibitory effect of phospholipids in this study is thought to be caused by other factors, such as topological features or the stiffness of the surface.

Previous studies have demonstrated the importance of the topological properties in the mediation of macrophage polarization. Ti with nanotubular surfaces induced M1 or M2 phenotypes, depending on the size of the nanostructure [17]. M2-inducing nanostructures enhanced the osteogenic differentiation of mesenchymal stem cells and osseointegration in mice models via macrophage polarization [26]. Macrophage polarization was also found to be modulated by the submicron-scale surface roughness of Ti [27]. Macrophage polarization is known to be accompanied by the change of cell shape. M1 and M2 macrophages exhibited different degrees of elongation [28], and it was found that intended elongation of cells by the micropatterning of surface induced M2 polarization [5]. Different degrees of nanopatterning influenced macrophage polarization differently [29]. Therefore, the topological properties are able to affect macrophage polarization via modulating cell shapes. In this study, the phospholipid coating changed the surface morphology of the titanium discs, which can affect the cell morphology and polarization of macrophages. Considering the relationship among topological features, cell shapes, and phenotypes, it is speculated that the dispersion of phospholipid structures interferes with macrophage activation by inhibiting cell elongation. Our results indicate that the inhibitory effect of the surface morphology exceeded the M2-inducing effect of phosphatidylserine. The other factor that we can consider is surface stiffness. Blakney et al. found that macrophages became less active on softer hydrogels [30]. The softer hydrogel resulted in less fibrosis in vivo. Macrophages on softer polyacrylamide also released smaller amounts of pro-inflammatory mediators when stimulated by LPS [31]. Considering the physical property of Ti and hydrated phospholipids, it is certain that hydrated phospholipids partly assigned softness to the Ti surface. As shown in Figure 1, the Ti surface was not completely covered with phospholipids. Therefore, macrophages should prefer phospholipids rather than Ti for the stiffness theory to be applied to our results. The preference of macrophages for phospholipids could not be confirmed by SEM images (Figure 2). However, the affinity between the PS of phospholipids and the PS receptor of macrophages was expected to facilitate cell adherence to the hydrated phospholipid structure. The translocation of TRITC-DHPE into cells in Figure 7 indicates that hydrated phospholipids can be readily associated with macrophages.

To enhance the biocompatibility of biomaterials, the M2 polarization of macrophages is generally recommended, because anti-inflammatory M2 macrophages are supposed to end inflammation and promote tissue regeneration. Meanwhile, the suppression of macrophage activation has been mostly studied in the pharmaceutical field to treat inflammatory diseases. In this study, macrophages were barely activated on phospholipid-coated Ti surfaces. Considering the central role of macrophages in tissue inflammation, it is expected that the suppression of macrophage activation will impede the inflammatory tissue response. Therefore, the phospholipid coating prepared in this study may attenuate inflammation while inhibiting the M2 polarization of macrophages. Although the in vivo effects of the phospholipid coating remain elusive, less inflammation and less active tissue regeneration is expected based on our in vitro results.

## 5. Conclusions

Various shapes of phospholipids (PS/PC) were formed on Ti discs through the hydration of dried phospholipids. When the concentration of loaded phospholipids was high (10 mM), a stable spherical structure was formed on Ti surface. The polarization of macrophages was disturbed on the phospholipid-coated surface, despite the presence of PS in phospholipids. The gene expression and protein release of TGF-β1, VEGF, Arg-1, and TNF-α in RAW 264.7 cells were downregulated, especially with 10 mM phospholipids. Although the underlying mechanism for the inhibitory effect of phospholipid coating is not yet fully understood, it is speculated that the topological feature of the coated surface inhibited macrophage polarization by interfering with the transformation of cell shape, e.g., degree of elongation. The other possibility is that the softened surface due to the phospholipid coating disturbed the polarizing activity of RAW 264.7 cells. The results of this study suggest that a method for phospholipid coating is an important factor for determining the cellular behavior of macrophages.
